# Maturation State and Matrix Microstructure Regulate Interstitial Cell Migration in Dense Connective Tissues

**DOI:** 10.1038/s41598-018-21212-4

**Published:** 2018-02-19

**Authors:** Feini Qu, Qing Li, Xiao Wang, Xuan Cao, Miltiadis H. Zgonis, John L. Esterhai, Vivek B. Shenoy, Lin Han, Robert L. Mauck

**Affiliations:** 1 McKay Orthopaedic Research Laboratory, Department of Orthopaedic Surgery, Perelman School of Medicine, University of Pennsylvania, Philadelphia, PA 19104 USA; 2 Translational Musculoskeletal Research Center, Corporal Michael J. Crescenz VA Medical Center, Philadelphia, PA 19104 USA; 3 Department of Bioengineering, University of Pennsylvania, Philadelphia, PA 19104 USA; 4 School of Biomedical Engineering, Science, and Health Systems, Drexel University, Philadelphia, PA 19104 USA; 5 Department of Materials Science and Engineering, University of Pennsylvania, Philadelphia, PA 19104 USA; 6 Center for Engineering MechanoBiology, University of Pennsylvania, Philadelphia, PA 19104 USA

## Abstract

Few regenerative approaches exist for the treatment of injuries to adult dense connective tissues. Compared to fetal tissues, adult connective tissues are hypocellular and show limited healing after injury. We hypothesized that robust repair can occur in fetal tissues with an immature extracellular matrix (ECM) that is conducive to cell migration, and that this process fails in adults due to the biophysical barriers imposed by the mature ECM. Using the knee meniscus as a platform, we evaluated the evolving micromechanics and microstructure of fetal and adult tissues, and interrogated the interstitial migratory capacity of adult meniscal cells through fetal and adult tissue microenvironments with or without partial enzymatic digestion. To integrate our findings, a computational model was implemented to determine how changing biophysical parameters impact cell migration through these dense networks. Our results show that the micromechanics and microstructure of the adult meniscus ECM sterically hinder cell mobility, and that modulation of these ECM attributes via an exogenous matrix-degrading enzyme permits migration through this otherwise impenetrable network. By addressing the inherent limitations to repair imposed by the mature ECM, these studies may define new clinical strategies to promote repair of damaged dense connective tissues in adults.

## Introduction

Dense connective tissues, such as the knee menisci, tendons and ligaments, and the annulus fibrosus of the intervertebral disc, are essential for the mechanical functionality of the musculoskeletal system. However, injuries often culminate in poor repair, leading to altered biomechanical function and eventually tissue and/or joint degeneration. Unfortunately, what little regenerative capacity exists also declines with tissue maturation. For example, fetal tissues exhibit a robust healing response^1–3^, and meniscal tears are rarely seen in children but are a common occurrence in adults^4,5^. Moreover, increasing patient age correlates with worse clinical outcomes after meniscal repair, including higher rates of repair failure^6,7^. Consequently, many clinical treatments focus on tissue removal rather than restoration, which temporarily alleviates pain but ultimately leads to irreversible deterioration of the affected joint. As such, strategies that enhance endogenous repair may benefit the aging population by delaying or even eliminating the need for end-stage total joint replacement.

Healing is characterized by cellular invasion into the wound site, with subsequent proliferation, synthesis of new matrix to bridge the wound gap, and tissue remodeling. A sufficient number of reparative cells at the wound interface is thus a critical early step in successful integrative repair. However, cellularity in dense connective tissues decreases progressively with age, with a very low cell density in the adult^1,4^. This deficiency in cell number may be compounded by the limited mobility of these cells through the physically restrictive microenvironment of adult tissue. During development, ECM collagen and proteoglycan (PG) content increase with load-bearing use, resulting in increased bulk mechanical properties^1^. Unlike migration in 2D (where increasing substrate stiffness generally increases migration speeds), adult cells in a 3D environment must overcome the increased biophysical resistance of their surrounding environment. As the pores through which cells crawl become progressively smaller and the matrix constituting the pore walls stiffens, migration rates decline and eventually cells are rendered immobile^8^. Thus, spatial confinement within the ECM may prevent endogenous cells from reaching the injury site to affect repair in adult dense connective tissues^9^.

Cells can partly overcome the steric hindrance of a dense and stiff microenvironment via cell deformation and/or matrix remodeling^10–12^. Ulrich and colleagues found that increasing the gel stiffness induces a mesenchymal-to-amoeboid transition in cell motility^13^. In particular, cells with compliant nuclei, such as leukocytes and certain neoplastic cells, remain highly mobile in tight interstices^8,10,14^. Introducing matrix metalloproteinase (MMP)-degradable linkages into stiff hydrogels can also enhance cell migration^15^. Conversely, cell mobility through small pores is further reduced when endogenous MMPs are inhibited^8^. Despite the wealth of knowledge gained from recent 3D migration studies, the vast majority of *in vitro* microenvironments, such as Matrigel^16^, collagen gels^8,17^, synthetic hydrogels^15^, or microfabricated chambers^18,19^, bear little resemblance to native dense connective tissues. Furthermore, the high level of collagen crosslinking and alignment in native tissues results in a tightly packed and organized fibrous network with increased resistance to proteolysis. Indeed, observations in isotropic, non-native environments likely do not recapitulate the impediments to migration experienced in dense connective tissues, and so there is a pressing need to develop new systems to study 3D cell migration in a more physiologic context.

To address this limitation, we investigated interstitial cell migration using devitalized tissue substrates as our experimental 3D milieu. We hypothesized that the native ECM is a biophysical impediment to cell mobility during repair, and that reduction of both steric and mechanical hindrances would expedite cell migration to the wound site. Using the adult knee meniscus as a test platform, we determined that age-related micromechanical and microstructural changes in the ECM are inhibitory to cell migration. Furthermore, we demonstrated that modulating ECM properties, via the application of exogenous matrix-degrading enzymes, enhanced interstitial mobility, and that this acted synergistically with cell-produced MMPs to promote cell migration through the dense ECM. These studies provide evidence of the role of native ECM properties on cell migration and establish new clinical strategies to promote endogenous repair of the meniscus and other dense connective tissues of the musculoskeletal system.

## Results

### ECM Microstructure and Micromechanics Change with Maturation State

**Figure 1:**
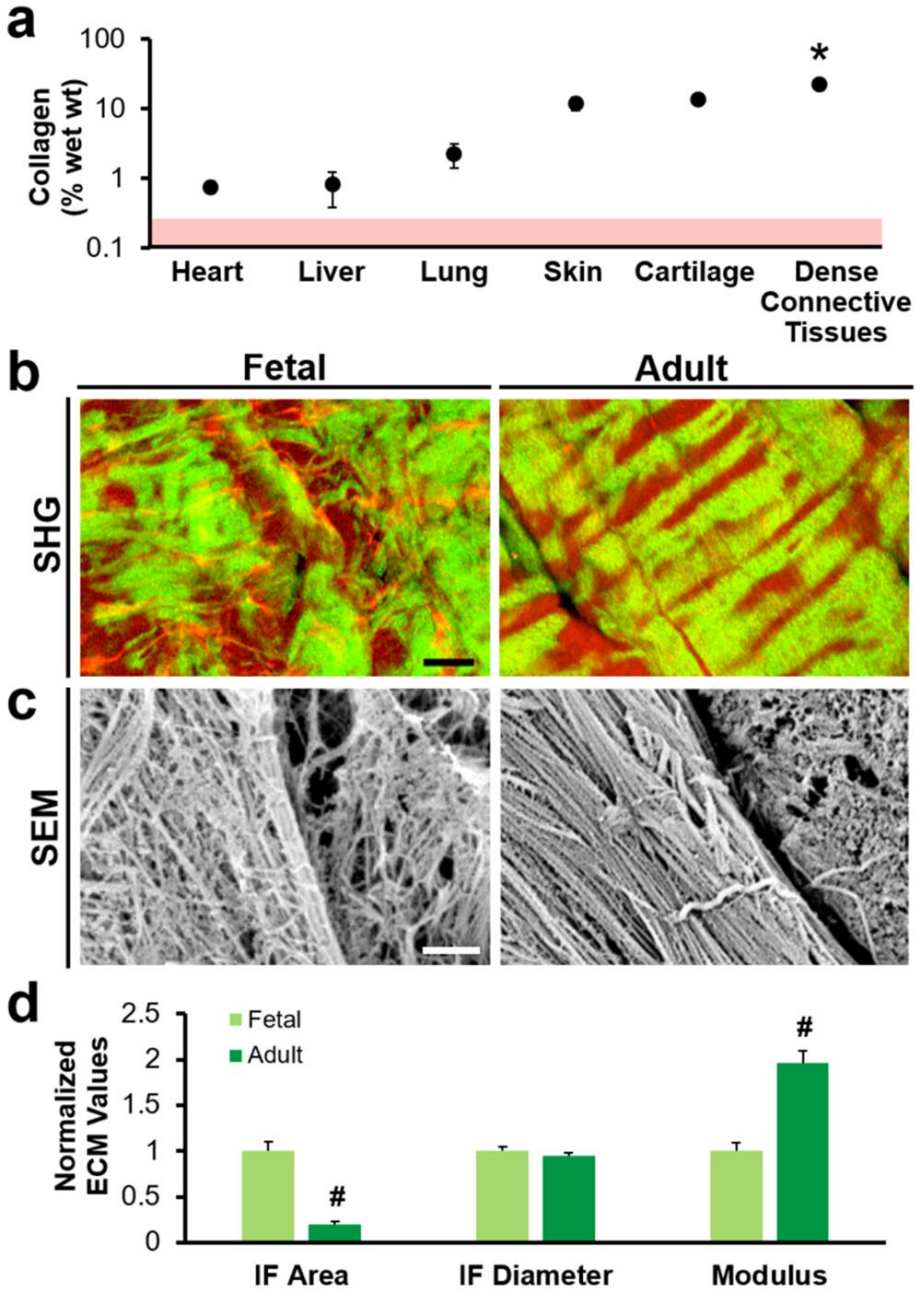
Collagen content and organization vary according to tissue type and maturation state. (**a**) Collagen content (% wet weight) of various mammalian tissues, shown on a semi-log plot (mean ± SD, *n* = 3–4 reference values per tissue type). Red area represents range of 3D collagen gels commonly used for *in vitro* migration studies. (**c**) Second harmonic generation (SHG) images of Fetal and Adult knee meniscus show circumferential collagen fibers, with composite SHG (green) and autofluorescent signal (red). Scale = 25 µm. (**d**) Scanning electron microscopy shows fibril alignment in a circumferential fiber bundle and inter-fibrillar discontinuities. (**d**) Fetal and Adult ECM values (normalized to Fetal ECM), including the area fraction and average diameter of inter-fibrillar regions (*n* = 10 samples/group, mean ± SEM) and tissue elastic modulus (measured via AFM indentation, *n* = 50 indentations/group, mean ± SEM). Scale = 1 µm. ^#^*p* < 0.05 vs. Fetal tissue. *p < 0.05 vs. all other groups.

As an initial survey of extracellular matrix (ECM) density, we compared the collagen content of various tissues from the literature. The mean collagen content of dense connective tissues (~21% wet wt) was significantly higher than that of other soft tissues (articular cartilage and skin) and internal organs (liver, heart, and lung) (*p* < 0.05, Fig. 1a and Supplementary Table S1). Importantly, dense connective tissues contained up to 100 times the concentration typically used for *in vitro* migration assays in collagen gels (~0.2% wet wt)^8,16–18,20^, suggesting that the interstitial spaces in native tissues are considerably less permissive to migration than in these *in vitro* preparations.

To directly query the impediments to cell migration in dense connective tissues, and how they might be affected by tissue maturation, we first inspected the microenvironment of the knee meniscus at two developmental states. Analysis of Fetal and Adult bovine meniscal tissue (~35 μm thick) revealed biophysical alterations in the ECM with age (Fig. 1b–d). Collagen fibers in Fetal tissue had poorly defined boundaries and appeared as a disorganized network (Fig. 1b,c). In contrast, collagen fibers in Adult tissue were thicker and appeared as distinct aligned bundles with patterned crimp. Areas of attenuated second harmonic generation (SHG) signal indicated discontinuities in the collagenous microstructure and corresponded either to collagen fibers in the perpendicular plane or other non-collagenous material in the ECM, such as proteoglycans (Supplementary Fig. S1). The area fraction of this inter-fibrillar ECM was lower for Adult tissue compared to Fetal tissue, and discrete inter-fibrillar areas were on average narrower in Adult tissue compared to Fetal tissue (*p* < 0.05, Fig. 1d). In addition, the local modulus of Adult tissue was approximately 2 times greater than that of Fetal tissue, most likely due to the well-organized fiber bundles (*p* < 0.05, Fig. 1d). Fiber density and organization in the orthogonal (radial) plane was similarly increased with maturation (Supplementary Fig. S2). Taken together, these data suggest that Adult tissue would be less amenable to cell invasion compared to Fetal tissue.

### Cell Morphology and Migration Are Dependent on ECM Properties

**Figure 2:**
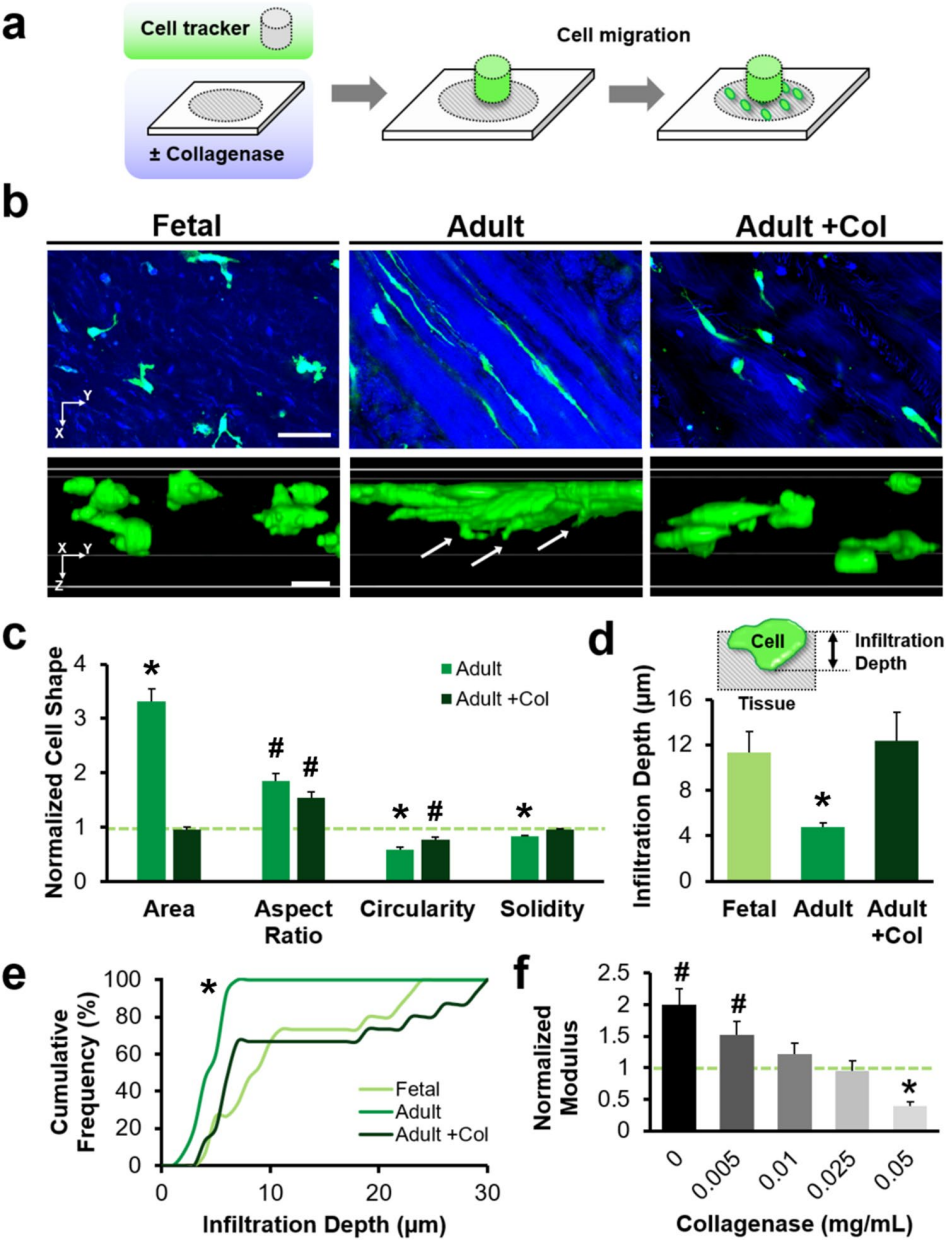
Cell morphology and interstitial mobility depend on tissue maturation state. (**a**) Experimental schematic of cell migration through devitalized meniscus sections. (**b**) Confocal images of cells migrating through Fetal, Adult, and Adult tissue treated with collagenase (Adult + Col). 2D slices and 3D reconstructions show cells (green) within the tissue depth (blue). Arrows indicate cellular protrusions. Scale = 50 µm. (**c**) 2D morphology of adult meniscal cells on Adult and Adult + Col substrates, normalized to Fetal values (dashed green line) (*n* = 71–97 cells/group, mean ± SEM). (**d**) Cell infiltration depth with schematic inset (*n* = 15 cells/group, mean ± SEM). (**e**) Cumulative frequency of infiltration depth. (**f**) Local indentation modulus of Adult tissues after graded collagenase digestion, normalized to Fetal tissue (green dashed line) (*n* = 19–20 indentations/group, mean ± SEM). ^#^*p* < 0.05 vs. Fetal tissue. *p < 0.05 vs. all other groups.

To test this hypothesis, we investigated the impact of tissue age and ECM structure on interstitial cell migration. To demonstrate that this was a matrix-autonomous effect, and not due to changes in cell function with maturation, the interstitial migration of cells derived from adult meniscus was investigated in the context of Fetal and Adult tissue, as well as Adult tissue that had been treated with collagenase to reduce its density and stiffness (Adult + Col) (Fig. 2a). Fluorescently labeled adult meniscus cells began infiltrating into devitalized substrates within 48 hours (Fig. 2b). Cell morphology on native tissue depended on the underlying ECM characteristics. That is, cells that attached to Adult tissue were 3× larger than they were on Fetal tissue (*p* < 0.05, Fig. 2c). Cells on both substrates aligned in the fiber direction of the underlying tissue, although cells on Adult tissue had a higher aspect ratio and exhibited lower circularity and solidity compared to cells on Fetal tissue (*p* < 0.05, Fig. 2c). Cells that had egressed onto partially digested Adult tissue (Adult + Col) were smaller and rounder compared to those on Adult tissue, more closely approximating the characteristics of adult cells interacting with Fetal tissue (*p* < 0.05, Fig. 2c). The migrating cells did not interact with the devitalized cells that had originally populated the tissue, which are indicated by DAPI-stained nuclei embedded within the substrate.

Cells also invaded the underlying substrate to differing extents. Those migrating through Adult tissue were more deformed compared to those migrating through Fetal and Adult + Col substrates, forming narrow protrusions into the surrounding matrix. Infiltration depth was greater for cells on Fetal tissue compared to cells on Adult tissue (*p* < 0.05, Fig. 2d). When the Adult tissue was digested, cells migrated to a similar extent as through Fetal tissue, with 20% of the population reaching depths of ≥25 µm from the surface (*p* < 0.05, Fig. 2e). The degree of matrix degradation was reflected by changes in the local modulus, which decreased with increasing collagenase dose (*p* < 0.05, Fig. 2f). Treatment with 0.05 mg/mL collagenase reduced the modulus of adult ECM to 8.6 ± 1.0 kPa, which was significantly lower than both untreated Fetal and Adult ECM (*p* < 0.05). Treatment with even higher doses of collagenase (0.1 mg/mL) further altered matrix microstructure, though these samples were not testable by AFM due to their dehiscence from the underlying slide during indentation.

### Dose-Dependent ECM Degradation Enhances Interstitial Cell Migration

**Figure 3:**
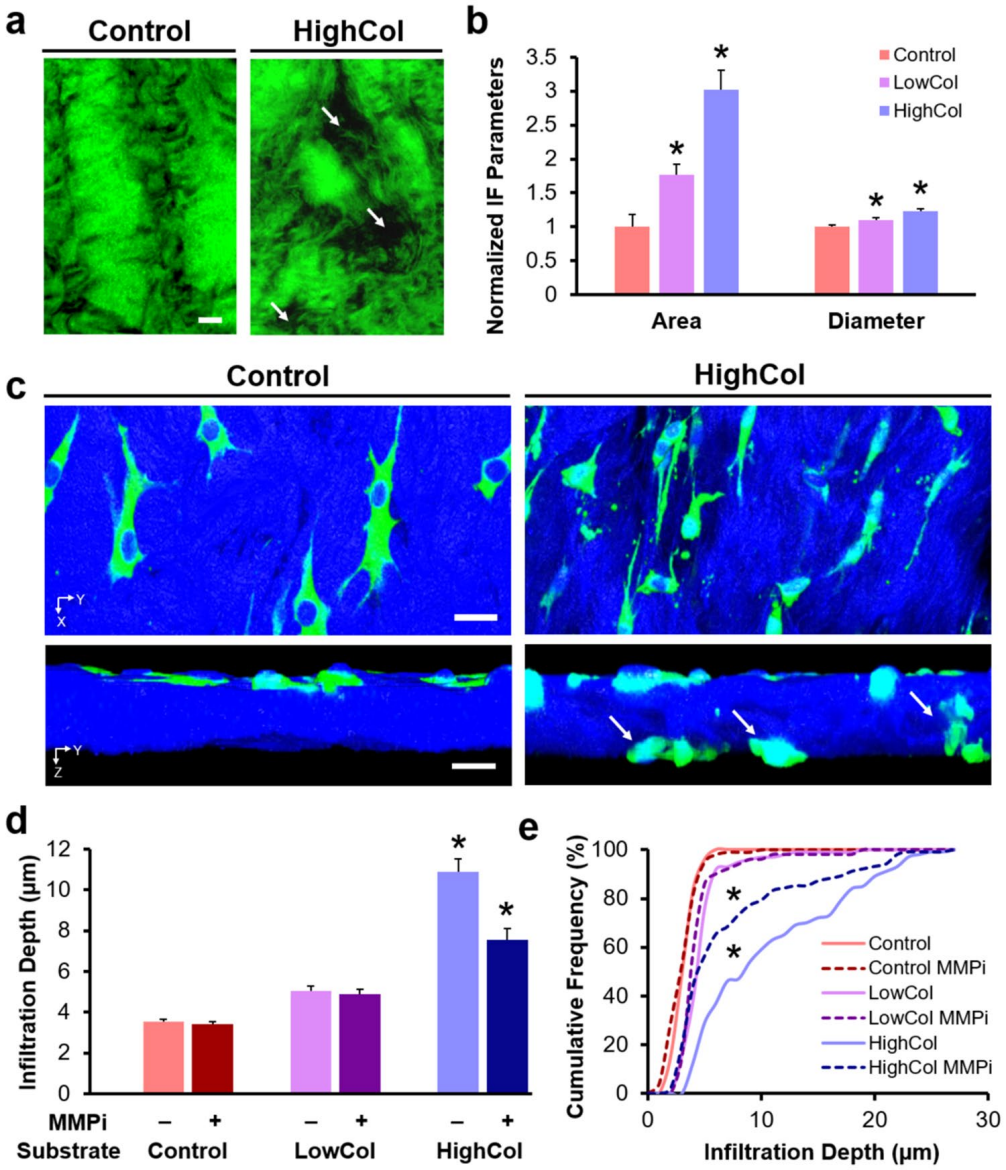
Exogenous and endogenous matrix degradation work synergistically to enhance cell migration. (**a**) SHG signal (green) of substrates showing altered fibrillar collagen structure with collagenase digestion. Arrows indicate inter-fibrillar regions. Scale = 20 µm. (**b**) Area fraction and average diameter of inter-fibrillar regions (*n* = 10 samples/group, mean ± SEM). (**c**) Top-down and cross-sectional 3D confocal reconstruction of adult meniscal cells (green) on tissue substrates (blue). Arrows point to infiltrating cells. Scale = 20 µm (top) and 10 µm (bottom). (**d**) Average and (**e**) cumulative frequency distribution of cell infiltration depth (*n* = 100 cells/group, mean ± SEM). *p < 0.05 vs. all other groups.

Next, to identify which of the biophysical features of the matrix most impact migration, we modulated the microenvironment independently, using two matrix-degrading enzymes: collagenase, which cleaves collagen and as a consequence releases collagen and proteoglycans from the ECM, and chondroitinase ABC (ChABC), which selectively degrades only chondroitin sulfate, a component of matrix proteoglycans. We also determined the extent to which cell-produced proteases contributed to the migration we observed. In addition to micromechanical changes, adult meniscal sections pre-treated with various levels of collagenase revealed distinct ECM morphologies (Fig. 3a). Qualitatively, untreated Control substrates had thicker and more organized collagen bundles than the low-dose (LowCol) and high-dose (HighCol) collagenase groups. The area fraction of inter-fibrillar ECM and the diameter of discrete inter-fibrillar regions increased with collagenase dose, suggesting local interruption of the native collagen network (*p* < 0.05, Fig. 3b).

As with the above studies, cells in the untreated Control group remained predominantly spread on the tissue surface, whereas cells in the collagenase groups were found within the tissue or had migrated completely through the tissue substrate (Fig. 3c and Supplementary Video S1). Cell infiltration was significantly greater for the higher levels of collagenase (HighCol) compared to lower levels of collagenase (LowCol and Control), with approximately half of the cells migrating ≥10 µm from the surface, and over 10% of cells reaching depths of ≥20 µm (*p* < 0.05, Fig. 3d,e). When matrix metalloproteinases (MMPs) produced by the cells were inhibited (MMPi), cell infiltration decreased for the HighCol group only. These findings reveal that cell-generated MMPs play a role in facilitating interstitial migration in this *in vitro* context, but that a certain degradative threshold must be reached before cell-mediated proteolysis can effectively promote migration through adult meniscal ECM.

**Figure 4:**
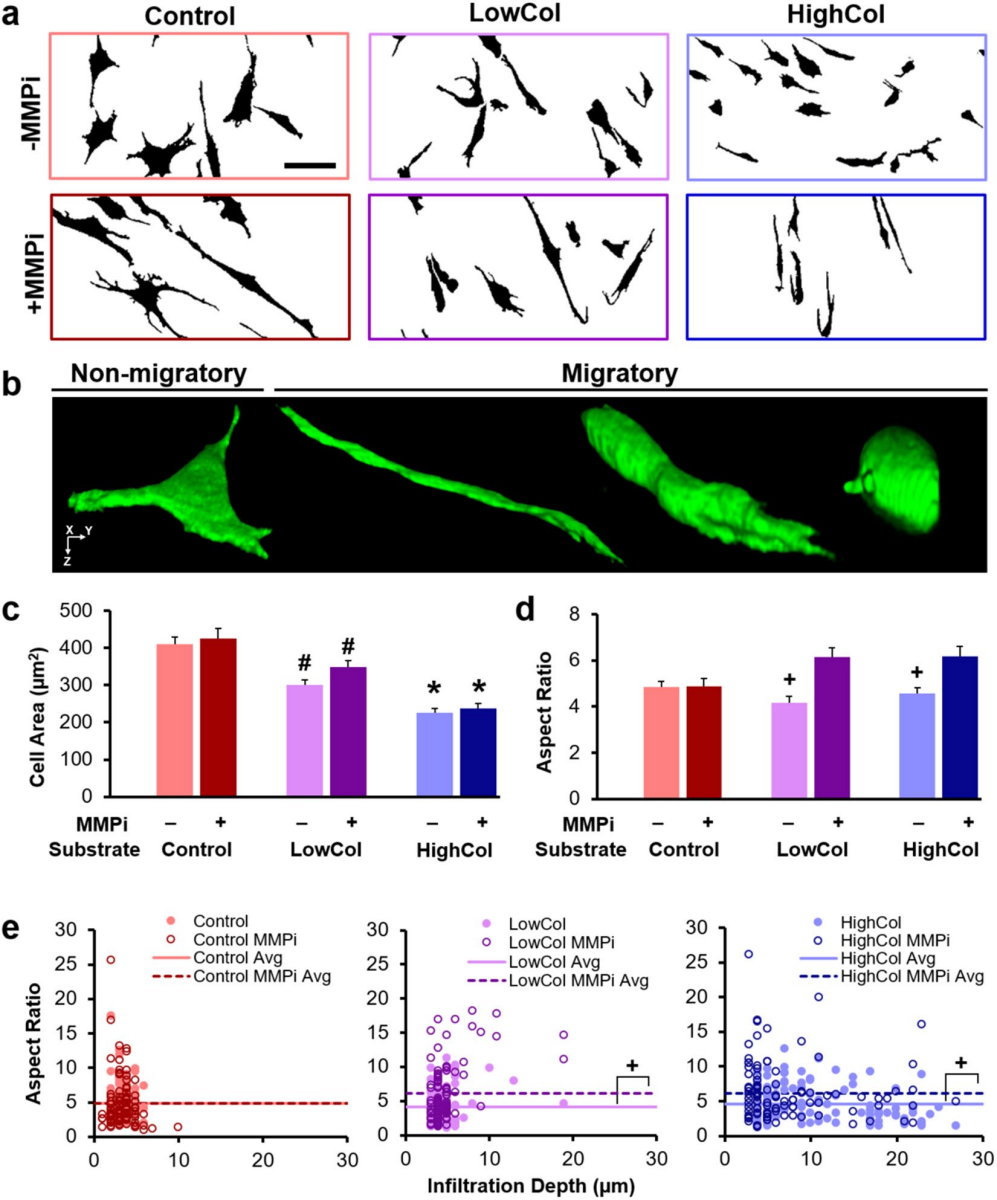
Migratory cell morphology is determined by exogenous and endogenous matrix degradation. (**a**) Binarized confocal maximum projections showing cells (black) on substrates of various degradative states with or without MMP inhibition (MMPi). Scale = 50 µm. (**b**) 3D confocal reconstruction of a non-migratory cell atop untreated tissue (Control), and migratory cells of various morphologies within collagenase-treated tissue (not shown to scale). (**c**) 2D cell area and (**d**) aspect ratio (*n* = 100 cells/group, mean ± SEM). (**e**) Aspect ratio as a function of infiltration depth (*n* = 100 cells/group). Scatter plot markers represent individual cells and lines represent group averages. Cells with infiltration depth ≥10 µm are considered highly infiltrative. ^#^*p* < 0.05 vs. Control, ^+^*p* < 0.05 vs. MMPi, *p < 0.05 vs. all other groups.

Cell morphology during migration also depended on the degree of substrate degradation and MMP inhibition (Fig. 4). The projected 2D cell area decreased with increasing collagenase dose (*p* < 0.05, Fig. 4c), reflecting fewer cells spread atop the tissue surface. Cell aspect ratio decreased with collagenase pre-treatment, suggesting that less deformation was required for cells migrating through a more compliant and porous tissue. Addition of MMPi increased the aspect ratio, especially for the subset of highly infiltrative cells on substrates with low levels of digestion (*p* < 0.05, Fig. 4d). Specifically, the cells in the LowCol MMPi group that reached depths of ≥10 µm into the tissue (4% of the cells) were extremely elongated, with an average aspect ratio that was 2 times greater than the population average (Fig. 4e). This difference was present but attenuated in the LowCol group and altogether absent in the HighCol substrate groups. Circularity and solidity also increased with substrate degradation in the absence of MMPi (*p* < 0.05, Supplementary Fig. S3). These data suggest that the degree of cell deformation required for effective migration is reduced once sufficient degradation of the dense adult ECM is achieved.

**Figure 5:**
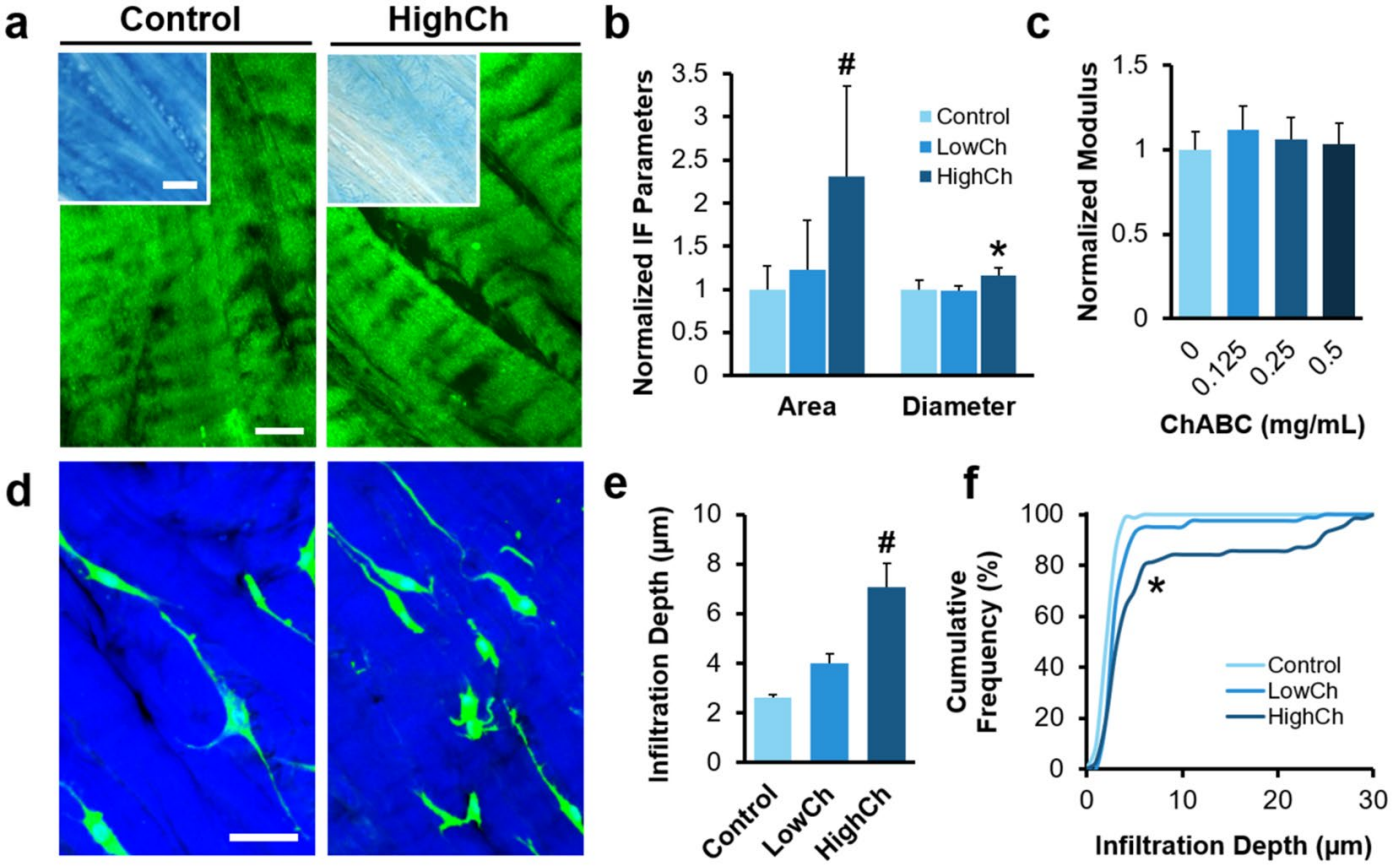
Modulating matrix microstructure in the absence of altered micromechanics improves cell migration. (**a**) SHG signal (green) of substrates showing fibrillar collagen structure with chondroitinase ABC (ChABC) digestion. Scale = 25 µm. Inset shows Alcian Blue staining of tissue proteoglycans. Scale = 100 µm. (**b**) Area fraction and average diameter of inter-fibrillar regions (*n* = 5 samples/group, mean ± SD). (**c**) Local indentation modulus of Adult tissues after digestion with varying concentrations of ChABC, normalized to untreated Control (*n* = 55–58 indentations/group, mean ± SEM). (**d**) Top down confocal images of adult meniscal cells (green) on tissue substrates (blue). Scale = 50 µm. (**e**) Average and (**f**) cumulative frequency distribution of infiltration depth (*n* = 70–80 cells/group, mean ± SEM). ^#^*p* < 0.05 vs. Control. *p < 0.05 vs. all other groups.

In contrast to our findings with collagenase, pre-treatment with ChABC to remove ECM proteoglycans did not substantially alter matrix microstructure and micromechanics (Fig. 5). While inter-fibrillar area fraction and diameter were marginally increased with high-dose ChABC (HighCh) treatment compared to the untreated Controls (*p* < 0.05, Fig. 5b), ChABC treatment did not alter the indentation modulus (*p* < 0.05, Fig. 5c). Despite these minor changes to the ECM microstructure, cell infiltration depth increased significantly in the HighCh group compared to the Control group (*p* < 0.05, Fig. 5e,f), although to a lesser extent than seen with collagenase digestion. The subset of migratory cells under this condition were not highly elongated, indicating that they were able to navigate through the enlarged inter-fibrillar regions that emerged after chondroitin sulfate removal (Supplementary Fig. S4). On the other hand, many highly elongated cells were unable to penetrate the matrix, suggesting that the stiff microenvironment remained a formidable barrier to cells that did not encounter sufficiently sized inter-fibrillar spaces. Collectively, these results demonstrate that both inter-fibrillar size and matrix stiffness are important rate-limiting factors to interstitial migration in dense connective tissues.

### ECM Properties Determine the Force Required for Cell Infiltration

**Figure 6:**
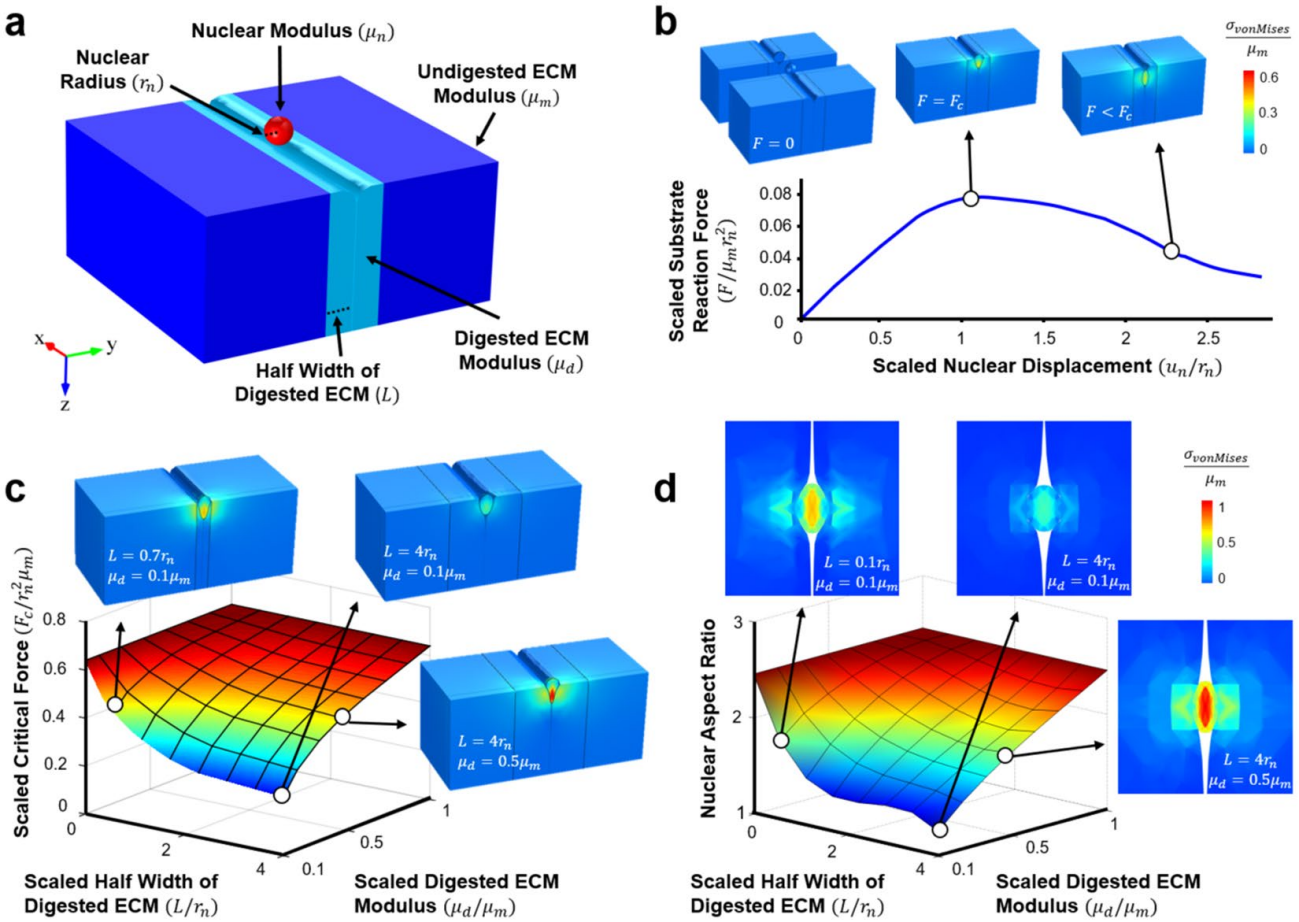
Computational model for cell migration through organized dense connective tissues. (**a**) Schematic showing a cell nucleus above a narrow slot in the ECM. The wall of the slot consists of a layer of digested ECM (light blue) that is softer than the undigested ECM outside (dark blue). (**b**) Simulation of a nucleus moving through a slot. The insets are contour plots showing the von-Mises stresses at the initial, critical, and migrated phases of cell entry. The scaled resistance force on the nucleus ($F/{\mu }_{m}{r}_{n}^{2}$) is plotted as as a function of the scaled vertical displacement of the nucleus (${u}_{n}/{r}_{n}$). (**c**) 3D heat map shows the scaled critical force (${F}_{c}/{r}_{n}^{2}{\mu }_{m}$) and (**d**) nuclear aspect ratio as a function of the scaled digested ECM modulus (${\mu }_{d}/{\mu }_{m}$) and the scaled half width of the digested ECM ($L/{r}_{n}$). Insets show the von-Mises stresses of the nucleus at the critical force and top down views of the nucleus within the slot.

To better understand the role of matrix parameters on cell migration, we developed a computational model to predict the critical force ($${F}_{c})$$ required for the nucleus of an invading cell (with radius $${r}_{n}$$ and stiffness $${\mu }_{n}$$) to navigate through a gap in the matrix (Fig. 6a). This model was motivated by studies of cellular extravasation in the context of cancer invasion, where the surrounding matrix properties (stiffness and pore size) appear to regulate this phenomenon^21^. Given our observation that cells and corresponding nuclei adopted an elongated morphology during migration in dense connective tissues, we simulated the topographic anisotropy of these highly aligned tissues by constraining cell migration through a narrow slot. The model also included two distinct matrix regions to mimic tissue degradation: a soft region located at the gap (degraded matrix with modulus $${\mu }_{d}$$) that spans a width of $$2L$$, and a stiff surrounding region (original matrix with modulus $${\mu }_{m}$$). Based on experimental values taken from this study and from previous work (*μ*_*m*_ = 50 kPa^22^, $${\mu }_{d}$$ = 25 kPa, $${\mu }_{n}$$ = 5 kPa^23^, $${r}_{n}$$ = 10 µm^24^, and $$L$$ = 20 µm), the model predicted a critical force of 370 nN (Fig. 6b). This critical force is of the same order of magnitude as that of the typical cytoskeletal traction force exerted by cells^25,26^.

As the stiffness of the degraded matrix (${\mu }_{d}$) increases, the critical force required for nuclear entry into a pore becomes greater (Fig. 6c). As this required force grows, it will eventually exceed the force generation capacity of the cytoskeleton, resulting in a situation where the cells can no longer infiltrate the substrate. Conversely, if ${\mu }_{d}$ decreases, cells can infiltrate, but they are forced to do so in an elongated manner where they must squeeze into the long and narrow channels between collagen bundles (Fig. 6d). In this case, most migrating cells have a high nuclear aspect ratio, consistent with our observations of highly elongated cells in the low-dose collagenase (LowCol) groups. If $L$ becomes very large and ${\mu }_{d}$ becomes very small, as would be the case with high levels of collagenase (HighCol), the model predicts that cells can infiltrate with ease and adopt either a rounded or elongated morphology, in agreement with our experimental observations (Fig. 4b).

## Discussion

Cell migration plays a pivotal role during tissue repair, where cells must first migrate to the defect, and then proliferate and form new matrix. Both high extracellular matrix (ECM) stiffness and density have been implicated as barriers to 3D migration^8,15^, especially when cell-mediated proteolysis via matrix metalloproteinases (MMPs) is inhibited. Although the highly organized collagen fibers of mature dense connective tissues enable mechanical function, they may inhibit cell migration after injury, resulting in poor healing in adults. Our findings suggest that cell mobility through dense connective tissues decreases with tissue maturation, which may be attributed to these emergent biophysical impediments in the adult microenvironment. Circumferentially aligned collagen fibers, the bulk constituent of the meniscal ECM, are thicker and more organized in adult tissue. Consequently, adult ECM is denser and stiffer on the microscale. These observations are consistent with previous histologic findings^1^, as well as other anatomical descriptions that span a wide range of imaging modalities^27–29^.

While enhanced microstructural organization, combined with higher collagen and proteoglycan content, improves the mechanical function of adult tissues, it does so at the expense of interstitial cell mobility. Importantly, infiltration occurred primarily at inter-fibrillar regions, which are more numerous and compliant in immature fetal tissue. Migrating cells appear rounded within fetal tissue, whereas cells migrating through adult tissue must deform through narrow crevices between rigid, aligned collagen bundles, similar to the steric constraints imposed by decreasing pore size in Transwell assays^8^ and microchannels^18^. Interestingly, cells do not prefer direct routes through the depth of adult tissues, which would force them to advance perpendicular to the circumferential bundles. Instead, highly infiltrative cells navigate downward with a gradual, sloped trajectory, such that the cell and nucleus remain elongated and aligned with the surrounding collagen fibers. Indeed, others have shown that cells preferentially align and migrate along fibers in collagen gels, and that migration speed increases with collagen alignment^20,30^. This behavior is not apparent in the low density and isotropic environments commonly employed in studies of interstitial cell migration *in vitro*, and highlights how ECM density and architecture of mature dense connective tissues influence the mode and efficiency of cell migration. In addition to biophysical properties, biochemical cues in the microenvironment, including integrin-binding proteins, cytokines, and growth factors, may also impact cell adhesion and migration^8,31^. For example, after tissue injury, pro-inflammatory cascades may stimulate cellular production of MMPs and up-regulation of integrins^32^, as well as alter cytoskeletal structure and cell mechanics^33^. Our system is an effective platform to study interstitial migration in native tissues, and may be further modified to include biochemical signals that mimic these developmental or disease states.

Since obstructed interstitial cell migration may prevent proper healing of the adult meniscus and other dense connective tissues, one strategy to promote repair may be to first free native cells from the matrix to facilitate migration to the wound site. Indeed, we found that interstitial cell mobility increased with localized matrix degradation. Cells on adult tissue sections pre-treated with collagenase were smaller, rounder, and more invasive than the same cells on untreated tissue, similar to cells on fetal tissue. Partial enzymatic digestion improved cell mobility by increasing the area fraction and size of inter-fibrillar regions (via cleavage of collagen and/or removal of PGs^34^) and also by decreasing the local ECM stiffness (in the case of collagenase). The softer, fetal-like matrix may also promote cell invasion by up-regulating endogenous MMP secretion and inducing the formation of protrusive structures called invadosomes^35^. In addition, denatured collagen fragments may influence cell migration by acting as chemoattractants^36^, by altering cell-substrate binding^31^, and/or by releasing matrix-bound growth factors into the microenvironment^37^. Although the collagenase and ChABC-treated substrates may not be directly comparable due to potential differences in matrix composition after enzymatic treatment, our data suggests that inter-fibrillar size correlates with the capacity of a cell to enter the tissue, and so is a good indicator of permissivity to migration. We recently showed that limited pore size and density similarly restrict meniscus cell infiltration into polymer nanofibrous networks^24^, supporting this conclusion, and are currently in the process of independently tuning nanofiber stiffness^38^ and porosity^39^ in biomaterial scaffolds to modulate cell migration with orthogonal control of these two attributes.

On a smaller scale, proteolytic remodeling of the pericellular matrix by cell-secreted enzymes may generate gaps to allow for cell passage^11^. Blocking cellular MMPs in our system increased cell elongation and decreased infiltration depth, suggesting that exogenous and endogenous MMPs act together to remodel the ECM during migration. However, this effect was only observed in the high-dose collagenase group, indicating that when the modulus is too high, endogenous MMPs are insufficient to enable migration, and cells instead rely more on cell and nuclear deformation through the inter-fibrillar clefts. Importantly, the nucleus is considered the rate-limiting organelle in migration due to its large size and stiffness, where high nuclear stiffness decreases migratory speed in confined spaces^8,10,14,18^. Since mesenchymal stem cell differentiation^40^ and increasing ECM stiffness^41,42^ are correlated with nuclear stiffening, it is plausible that cell mobility in adult dense connective tissues may also be affected by biophysical changes to the nucleus. Indeed, the premature aging disorder Hutchinson-Gilford Progeria Syndrome is characterized by stiffer nuclei and decreased cell mobility in tight interstices^43^.

In summary, our results suggest that a critical level of matrix degradation is required for interstitial cell migration in adult dense connective tissues after injury. This may be especially relevant in the context of aged and/or degenerate menisci in osteoarthritic tissues, which exhibit increased micromechanical heterogeneity than younger human menisci^44^. However, if delivery of proteolytic enzymes is to achieve therapeutic relevance, treatment must be targeted to the defect to advance cell migration locally while preventing broader structural and mechanical damage to the meniscus and adjacent tissues. To that end, we developed a nanofibrous system that delivers a controlled release of collagenase directly to the wound site^34^. Previously, we showed that reprogramming the meniscal wound interface with collagenase reduces local ECM density and stiffness, increases interfacial cellularity, and facilitates repair^24,45^. Here, we provide evidence that expedited migration to the injury site is a major mechanism underlying these superior healing responses. Once cells have reached their destination, additional cues may be introduced to stimulate matrix deposition^46^, prevent matrix catabolism^47^, and promote collagen crosslinking^48^ and alignment^49^. By combining these diverse but complementary processes, the standard treatment paradigm for dense connective tissue injuries may soon shift from resection and replacement to preservation and repair.

## Materials and Methods

### Collagen Content Analysis

The mean collagen content (% wet wt) of various mammalian tissues was derived from the literature^50–61^ (Supplementary Table S1, *n* = 3–4 references values per tissue type). Hydroxyproline content was converted to total collagen using a multiplicative factor of 7.5^50^.

### Tissue Substrate Fabrication and Characterization

Fetal (late 2^nd^–3^rd^ trimester) and adult (20–30 months) bovine stifle joints were sterilely dissected and the knee menisci isolated. Cylindrical tissue explants (8 mm diameter) were excised from the central red/white zone of medial meniscal bodies and embedded in Optimal Cutting Temperature sectioning medium (OCT; Sakura Finetek, Torrance, CA). Samples were axially cut into ~35 µm thick sections onto glass slides using a cryostat microtome (Microm HM500; MICROM International GmbH, Waldorf, Germany), such that the predominant fiber direction was parallel with the slide surface (sections in the orthogonal plane were also generated for imaging).

Second harmonic generation (SHG) imaging was utilized to visualize fibrillar collagen bundles in Fetal and Adult tissue sections (*n* = 4 samples/group). Beam scanning was performed using a Zeiss LSM 510 NLO/META with a Zeiss Axiovert 200 M inverted microscope at 20X magnification (Carl Zeiss Microscopy GmbH, Jena, Germany). A tunable 1 W coherent Chameleon laser with a 90 MHz repetition rate and pulse width of <200 femtoseconds was used to generate an excitation wavelength of 840 nm. SHG and autofluorescent signals were separated into different channels using bandpass filters of 390–465 and 500–550 nm, respectively. *Z*-stacks at 1 µm intervals were acquired through the entire tissue thickness and analyzed via the open-source platform Fiji^62^. Maximum *z*-stack projections were converted into binary images to identify areas of positive and negative SHG signal, representing organized fiber bundles parallel to the substrate and inter-fibrillar material that constitutes the remaining ECM. Analysis focused on *z*-stacks that contained primarily circumferential fibers, and those with large areas of perpendicular radial fibers were excluded. Inter-fibrillar area fraction was calculated as a percentage of the total area. The average diameter of discrete inter-fibrillar regions was quantified via the Local Thickness plugin^63^.

Scanning electron microscopy (SEM) was used to make qualitative assessments of ECM organization at the nanoscale^64^. Cryosections were fixed with Karnovsky’s fixative (Electron Microscopy Sciences, Hatfield, PA) for 3 hours at room temperature. Samples were dehydrated in graded ethanol-water mixtures (ethanol volume ratio: 25%, 50%, 75%, 80% and 100%), followed by immersion in graded mixtures of hexamethyldisilazane (HMDS; Sigma-Aldrich) and ethanol (HMDS volume ratio: 25%, 50%, 75% and 100%). Samples were air dried overnight and sputter coated with platinum prior to imaging. Micrographs were taken under high vacuum with a 3 kV electron beam using a Supra 50VP SEM (Zeiss, Jena, Germany).

To assess tissue micromechanical properties as a function of age, nanoindentation was performed on hydrated Fetal and Adult tissue sections using an atomic force microscope (AFM). Cryotomed sections on glass slides were rinsed in phosphate buffered saline (PBS) to remove residual OCT compound. Force spectroscopy was conducted in PBS with a Dimension Icon AFM (Bruker, Billerica, MA), using a colloidal spherical tip (R ≈ 5 µm, nominal k ≈ 0.6 N/m)^22^. Indentation force as a function of depth into the tissue was measured as the microspherical tip indented the sample at a rate of 10 µm/s (*n* = 50 indentations/group for 3 samples). Effective indentation modulus (E_ind_) was calculated using a finite thickness-corrected Hertz model^65^ with a Poisson’s ratio (ʋ) of 0.01^66^ and a 35 µm nominal sample thickness via a custom MATLAB program (MathWorks, Inc., Natick, MA).

### Cell Egress and Invasion into Tissue Substrates

Cell infiltration of devitalized tissue substrates was investigated using Fetal and Adult meniscal cryosections, which were UV sterilized for 1 hour and triple-rinsed in PBS to remove residual OCT compound. To assess the effect of tissue age on interstitial cell mobility and to determine whether the matrix can be reprogrammed to influence migration, 3 substrate conditions were tested in a first study (*n* = 4 samples/group): Fetal, Adult, and Adult tissue pre-treated with basal media (BM; Dulbecco’s Modified Eagle’s Medium (DMEM) with 10% Fetal Bovine Serum (FBS) and 1% Penicillin/Streptomycin/Fungizone (PSF)) supplemented with 0.1 mg/mL collagenase (type IV from *Clostridium histolyticum*, ≥125 collagenase digestion units/mg solid; Sigma-Aldrich, St. Louis, MO) for 1 hour (Adult + Col). To obtain cells unaltered by expansion on tissue culture plastic, adult explants (5 mm diameter, 3 mm height) from the medial meniscus were incubated in BM. After ~3 weeks of *in vitro* culture, explants were rinsed in PBS and incubated in 5 µg/mL of 5-chloromethylfluorescein diacetate (CellTracker™ Green; Thermo Fisher Scientific Inc., Waltham, MA) in serum-free media (DMEM with 1% PSF) for 1 hour to fluorescently label cells lining the explant surface. Explants were maintained in serum-free media for an additional 30 minutes prior to being placed atop tissue substrates to allow for cell egress. Slides with explants were placed in 4-well plates, covered with BM, and cultured at 37 °C. After 48 hours in BM, explants were removed from the substrate, and slides were fixed with 4% paraformaldehyde. Samples were stained with 4′,6-diamidino-2-phenylindole (DAPI, Prolong Gold; Invitrogen, Grand Island, NY) to visualize cell nuclei.

Confocal *z*-stacks at 20X magnification and 1 µm intervals were obtained in the FITC and DAPI channels to visualize cells, nuclei, and devitalized matrix (autofluorescent in the DAPI channel) using a Nikon A1 confocal microscope (Nikon Instruments; Melville, NY). To edge detect cell and tissue boundaries respectively, *z*-stacks in the FITC (cell) and DAPI (tissue) channels were binarized using Fiji. A region of interest (ROI) circumscribing each cell was derived from the FITC maximum *z*-projection and used to calculate the following morphometric parameters: aspect ratio (ratio of the largest diameter and the smallest diameter orthogonal to it), circularity (4π*area*perimeter^−2^), and solidity (area/convex area) (*n* = 71–97 cells/group). Interstitial migration was assessed by quantifying infiltration depth, defined as the distance between the apical surface of the tissue and basal surface of the cell (*n* = 15 cells/group). A signal intensity profile in the *z* dimension was generated for each cell, where 0 and 255 indicated 0% and 100% area with positive signal within the ROI. The apical surface was defined as the *z* location where the ROI first reached 127.5 (50% positive signal), and the basal surface was defined as the last z location before the signal dropped below 127.5. Cells located within or near substrate defects (i.e., tears within the tissue) were excluded from analysis.

### Enzymatic Degradation of Tissue Substrates

To determine the effect of matrix degradation on the tissue microenvironment and interstitial migration, adult ECM of varying degraded states were generated by incubation with either collagenase or chondroitinase ABC (ChABC) in two separate studies. For the collagenase study, 3 groups were tested: untreated adult ECM (Control), and adult ECM pre-treated with 0.05 or 0.1 mg/mL of collagenase in BM for 1 hour (LowCol or HighCol). Adult meniscal explants were fluorescently labeled and placed atop substrates to allow for cell egress as previously described. To assess the contribution of endogenous, cell-produced matrix-degrading enzymes on migration, 2 media conditions were tested for each group (*n* = 4 samples/group): BM with or without 1 µg/mL of the broad spectrum MMP inhibitor GM6001 (MMPi; EMD Millipore Corporation, Billerica, MA). After 48 hours of incubation, samples were processed as in the previous study, and cell morphology and infiltration depth were quantified (*n* = 100 cells/group). To evaluate tissue microstructure after collagenase treatment, the inter-fibrillar area fraction and average diameter were quantified as previously described (*n* = 10 samples/group). Lastly, to assess changes in tissue micromechanics, AFM indentation was performed on a separate set of adult ECM substrates as previously described. Samples were incubated in BM with varying concentrations of collagenase for 30 minutes prior micromechanical evaluation (*n* = 19–20 indentations/group for 3 samples): 0 (Control), 0.005, 0.01, 0.025, or 0.05 mg/mL. To isolate the effect of proteoglycan removal from the matrix, 3 additional groups were tested: untreated adult ECM (Control), and adult ECM pre-treated overnight with 0.125 or 0.25 mg/mL of ChABC (LowCh and HighCh; Sigma-Aldrich) in a Tris buffer (50 mM Tris, pH 8.0, with 60 mM sodium acetate and 0.02% BSA) (*n* = 4 samples/group). Interstitial migration was assessed in BM for 48 hours, and the cells and tissues were analyzed as previously described (*n* = 70–80 cells/group). A separate set of substrates with the same treatment was used for micromechanical evaluation (*n* = 55–58 indentations/group for 3 samples). Afterwards, samples were stained with Alcian Blue to assess proteoglycan content^34^.

### Cell Migration Model

A computational model was developed to elucidate the role of matrix microstructure and micromechanics on cell migration. Infiltration was modeled as a spherical nucleus that is pulled into the matrix through a narrow gap between digested collagen bundles. The digested region was modeled as a straight band that extended infinitely in the longitudinal and substrate thickness directions, surrounded by a matrix with a fixed elastic modulus. The force required to pull the nucleus into the substrate and the substrate reaction force during nuclear displacement were estimated using an incompressible neo-Hookean model using COMSOL 4.4b (COMSOL Inc., Stockholm, Sweden). The size and stiffness of the digested matrix were varied to determine how these parameters influenced the force and nuclear shape required for cell entry into the substrate. Details of the model can be found in the Supplementary Methods.

### Statistical Analyses

All experiments were performed for 3 meniscal donors per condition. Statistical analyses were performed using SYSTAT (Systat Software, Inc., San Jose, CA). Significance was assessed by one or two-way ANOVA with Tukey’s HSD post hoc for collagen content, matrix stiffness, inter-fibrillar area fraction and average diameter, cell area and morphometric descriptors, and infiltration depth (*p* < 0.05). A cumulative distribution plot, coupled with the Kolmogorov-Smirnov test, was used to determine whether the distribution of infiltration was different between groups (*p* < 0.05). Independent variables include tissue age, substrate treatment, and media condition. Data are presented as mean ± standard error of the mean unless specified otherwise.

### Data Availability

All relevant data are available from the corresponding author.

## Electronic supplementary material

Supplementary Information

Supplementary Video 1
