# Supplementary material for: Maturation State and Matrix Microstructure Regulate Interstitial Cell Migration in Dense Connective Tissues
Source: Sci Rep. 2018 Feb 19;8:3295. doi: 10.1038/s41598-018-21212-4 (PMC5818574; doi:10.1038/s41598-018-21212-4)
Supplement: Supplementary file 1 — Supplementary Information [file 41598_2018_21212_MOESM1_ESM.pdf]

Maturation State and Matrix Microstructure Regulate Interstitial Cell Migration in Dense Connective Tissues

Feini Qu, Qing Li, Xiao Wang, Xuan Cao, Miltiadis H. Zgonis, John L. Esterhai, Vivek B. Shenoy, Lin Han, *Robert L. Mauck

Supplementary Methods

Cell Migration Model

To understand the role of nuclear deformation in cell infiltration into dense connective tissues, we considered the case of a substrate containing aligned collagen bundles. Infiltration occurs as a spherical nucleus of radius r_n is pulled into the matrix through the small gap between the digested collagen bundles. To reflect the microstructure of the digested substrate in our experiment, the digested region of the substrate was modeled as a straight band with a half width L and extends infinitely in both the tissue depth and longitudinal direction. In the center of the digested band, a narrow slot representing the gap between the digested collagen bundles was introduced. The slot ran parallel to the band, and had a width much smaller than the radius of the nucleus. The mechanical responses of both the nucleus and the matrix were computed using an incompressible neo-Hookean model, which accounts for the geometric nonlinearity due to large strain deformation. The Cauchy stress is given by: $\sigma_{ij} = \mu\left(F_{iK}F_{jK} - F_{mK}F_{mK}/3\right)$, where $F_{ij} = \partial x_i/\partial X_K$ is the deformation gradient, with the position vector to a material point in the initial and current configuration given by x_i and X_K, respectively. Here, μ is the shear modulus of the material, and we assigned μ_n, μ_m and μ_d to the nucleus, the undigested substrate, and the digested substrate, respectively. Since collagenase digestion causes softening of the matrix, μ_d decreases with increasing levels of treatment.

In the initial configuration, the nucleus was positioned on the surface of the substrate over the slot. We assigned the frictionless contact as the boundary condition between the nucleus surface and the slot wall, which allowed the nucleus and the slot wall to slide freely without penetrating one another. The nucleus was pulled into the slot by an evenly distributed stress σ applied on the bottom side of the nucleus. Under mechanical equilibrium, the substrate exerted a reaction force F that has the magnitude equal to the active force applied on the nucleus ($F = \int \sigma_{ij} n_j dA$, where n_j is the unit vector in the normal direction of the nucleus surface). The applied force represented the cytoskeletal contraction that deformed the nucleus and widened the surrounding slot to allow the cell to fit into the substrate, and the substrate reaction force was the resistance force that acted against the insertion of the nucleus. To calculate the deformation of the nucleus and the substrate during the insertion process, we implemented the model and the constitutive equation for Cauchy stress in the finite element package COMSOL 4.4b (COMSOL, Inc., Stockholm, Sweden). The simulation increased the applied stress and monitored the resistance force F as a function of the nuclear displacement, which was represented by displacement of the bottom of the nucleus. Besides the nuclear displacement, the resistance force F depended on the model parameters: (1) μ_n and r_n - elastic modulus and radius of the nucleus, (2) μ_m - modulus of the undigested (stiff) matrix, (3) μ_d - modulus of the digested (soft) matrix that surrounds the slot, and (4) L - half width of the digested region. This was simplified using a dimensionless function: $\tilde{F} = \frac{F}{\mu_m r_n^2} = \tilde{F}(\frac{u_n}{r_n}, \frac{L}{r_n}, \frac{\mu_d}{\mu_m}, \frac{\mu_n}{\mu_m})$, where we scaled all length parameters to the nuclear radius r_n and scaled all modulus parameters to the modulus of the undigested matrix μ_m.

Supplementary Figures

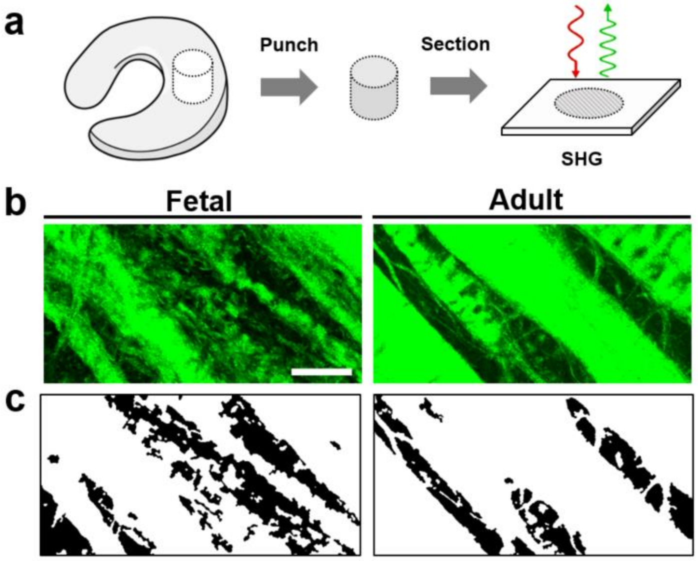

Supplementary Figure S1. The adult meniscal microenvironment is denser than that of fetal tissue. (**a**) Schematic showing meniscal sample preparation and second harmonic generation (SHG) imaging of tissue substrates. (**b**) SHG signal (green) showing the circumferential collage fibers of Fetal and Adult tissue. Scale = 50 µm. (**c**) Binarized images depicting inter-fibrillar areas in black.

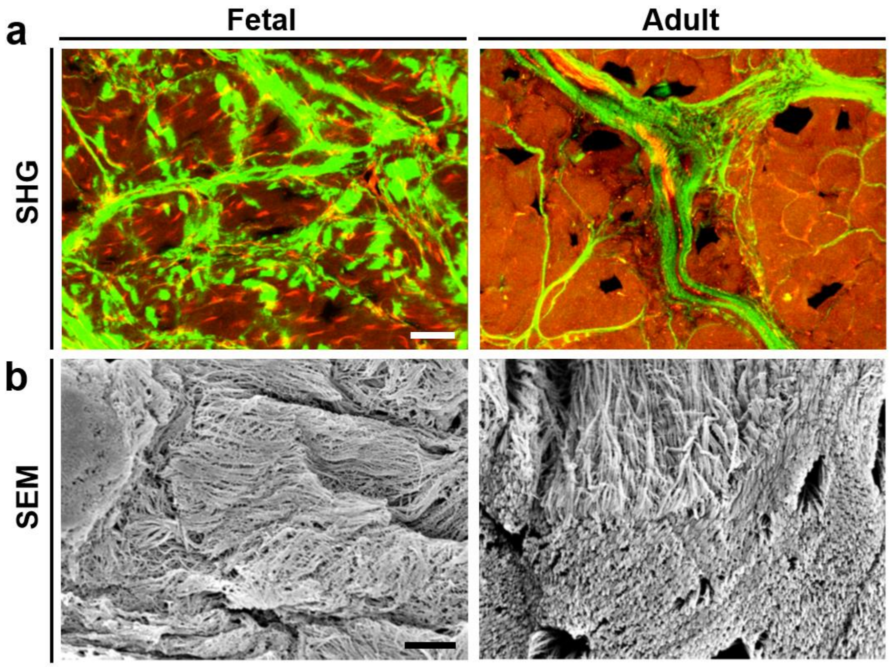

Supplementary Figure S2. Meniscal collagen fiber organization increases with maturation in the radial plane. (**a**) Images of Fetal and Adult tissue sectioned in the radial plane with composite SHG (green) and autofluorescent signal (red). Scale = 50 µm. (**b**) SEM micrographs of radially sectioned substrates. Scale = 2 µm.

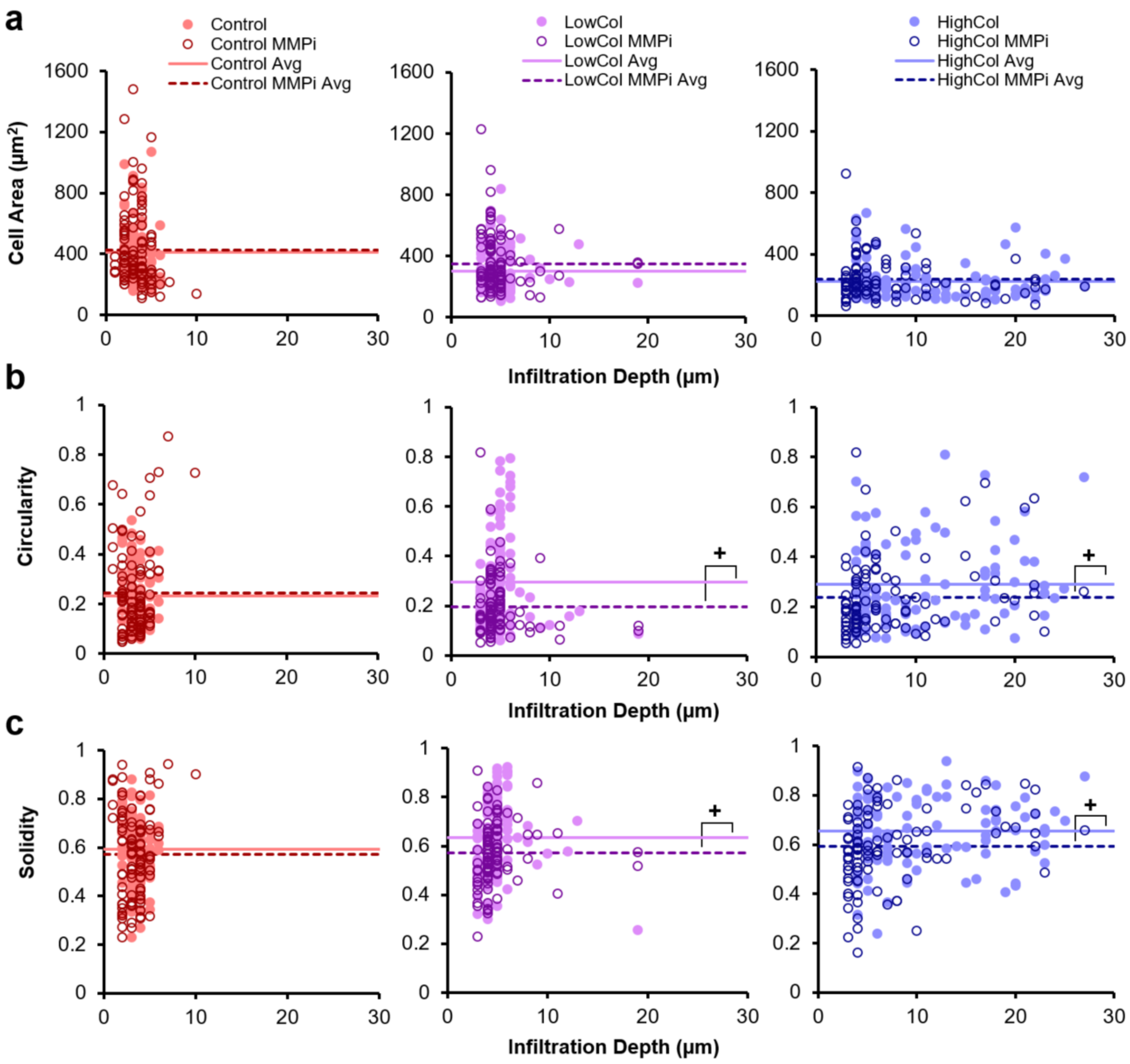

Supplementary Figure S3. Cell morphology vs. infiltration depth for varying collagenase treatments. (**a**) Cell area and morphometric values, including (**b**) circularity and (**c**) solidity, calculated from *z*-projections (*n* = 100 cells/group). Solid scatter plot markers represent individual cells and lines represent group averages for untreated (Control) and low- (LowCol) and high-dose collagenase (HighCol) substrate groups. Circles and dashed lines represent individual cells and group averages for substrate groups where the cell culture medium was supplemented with a MMP inhibitor (MMPi). Cells with infiltration depth ≥10 μm are considered highly infiltrative. + = $p<0.05$ vs. MMPi.

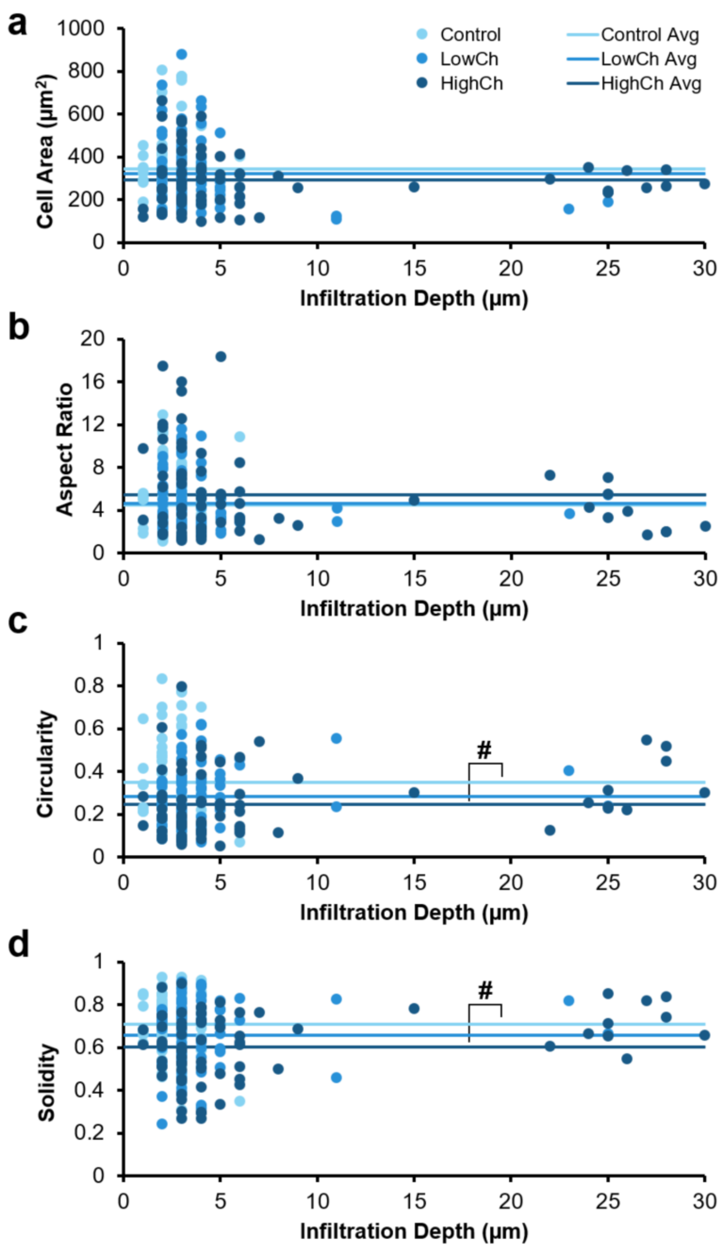

Supplementary Figure S4. Cell morphology vs. infiltration depth for varying chondroitinase ABC treatments. (**a**) Cell area and morphometric values, including (**b**) aspect ratio, (**c**) circularity, and (**d**) solidity, were calculated from *z*-projections (*n* = 70–80 cells/group). Scatter plot markers represent individual cells and lines represent group averages for untreated (Control) and low- (LowCh) and high-dose chondroitinase ABC (HighCh) substrate groups. Cells with infiltration depth ≥10 µm are considered highly infiltrative. # = *p*<0.05 vs. Control.

Supplementary Table

Tissue Type	Source	Collagen Content (Wet Wt%)
Liver	(a) Rat[51]<br>(b) Human[51]<br>(c) Human[50]	(a) 0.34<br>(b) 0.96<br>(c) 1.17
Heart	(a) Human[52]<br>(b) Human[50]<br>(c) Human, left ventricle[51]<br>(d) Human[53]	(a) 0.61<br>(b) 0.75<br>(c) 0.91<br>(d) 0.98
Lung	(a) Rat[54]<br>(b) Human[55]<br>(c) Human[50]	(a) 1.65<br>(b) 1.9<br>(c) 3.24
Skin	(a) Human[50]<br>(b) Human[56]<br>(c) Human[57]	(a) 10.05<br>(b) 11.3<br>(c) 15.0
Articular Cartilage	(a) Bovine, femur[58]<br>(b) Human, femur[59]<br>(c) Human, femur[60]	(a) 14.55<br>(b) 12.55<br>(c) 13.9
Dense Connective Tissues	(a) Bovine, medial collateral ligament[61]<br>(b) Bovine, patellar ligament[61]<br>(c) Bovine, medial meniscus[61]<br>(d) Human, tendon[50]	(a) 19.4<br>(b) 21.2<br>(c) 22.6<br>(d) 20.31

Supplementary Table S1. Mean collagen content (wet wt%) of mammalian tissues. Values derived from the literature (*n* = 3–4 references values per tissue type) illustrate the difference in collagen content for various organs and load-bearing musculoskeletal tissues.

Supplementary Video

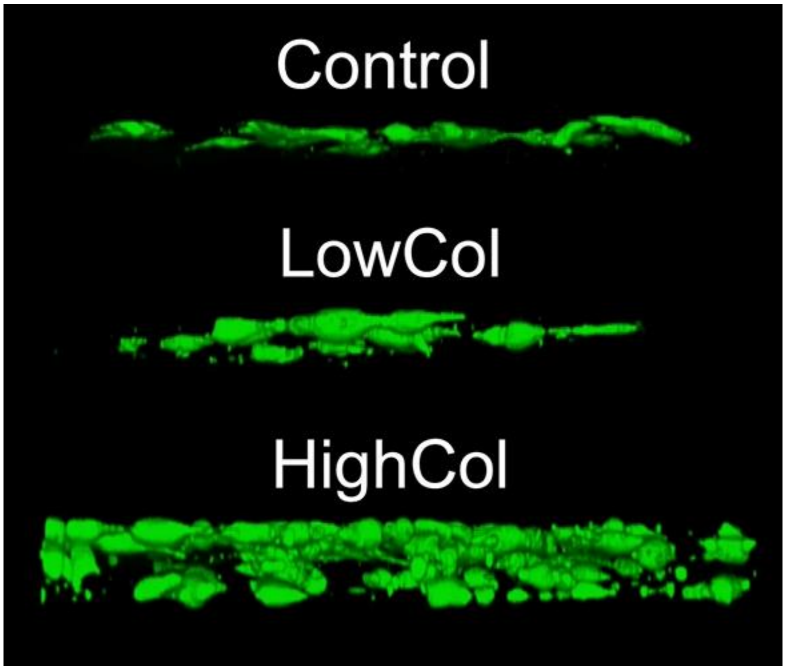

Supplementary Video S1. Cell migration through adult meniscal tissue is enhanced with partial matrix degradation. Top-down and cross-sectional 3D confocal reconstruction of adult meniscal cells (green) on tissue substrates (blue) for three treatment groups in the following order: no enzyme (Control), low-dose collagenase (LowCol), and high-dose collagenase (HighCol). Final montage compares cross-sectional views of cells from all three groups.
